# Supplementary material for: Beyond IbMYB1: Identification and Characterization of Two Additional Anthocyanin MYB Activators, IbMYB2 and IbMYB3, in Sweetpotato
Source: Plants (Basel). 2025 Sep 18;14(18):2896. doi: 10.3390/plants14182896 (PMC12473997; doi:10.3390/plants14182896)
Supplement: Supplementary file 1 [file plants-14-02896-s001.zip › plants-3838410 -Sup Figure-revised.pdf]

Figure S1

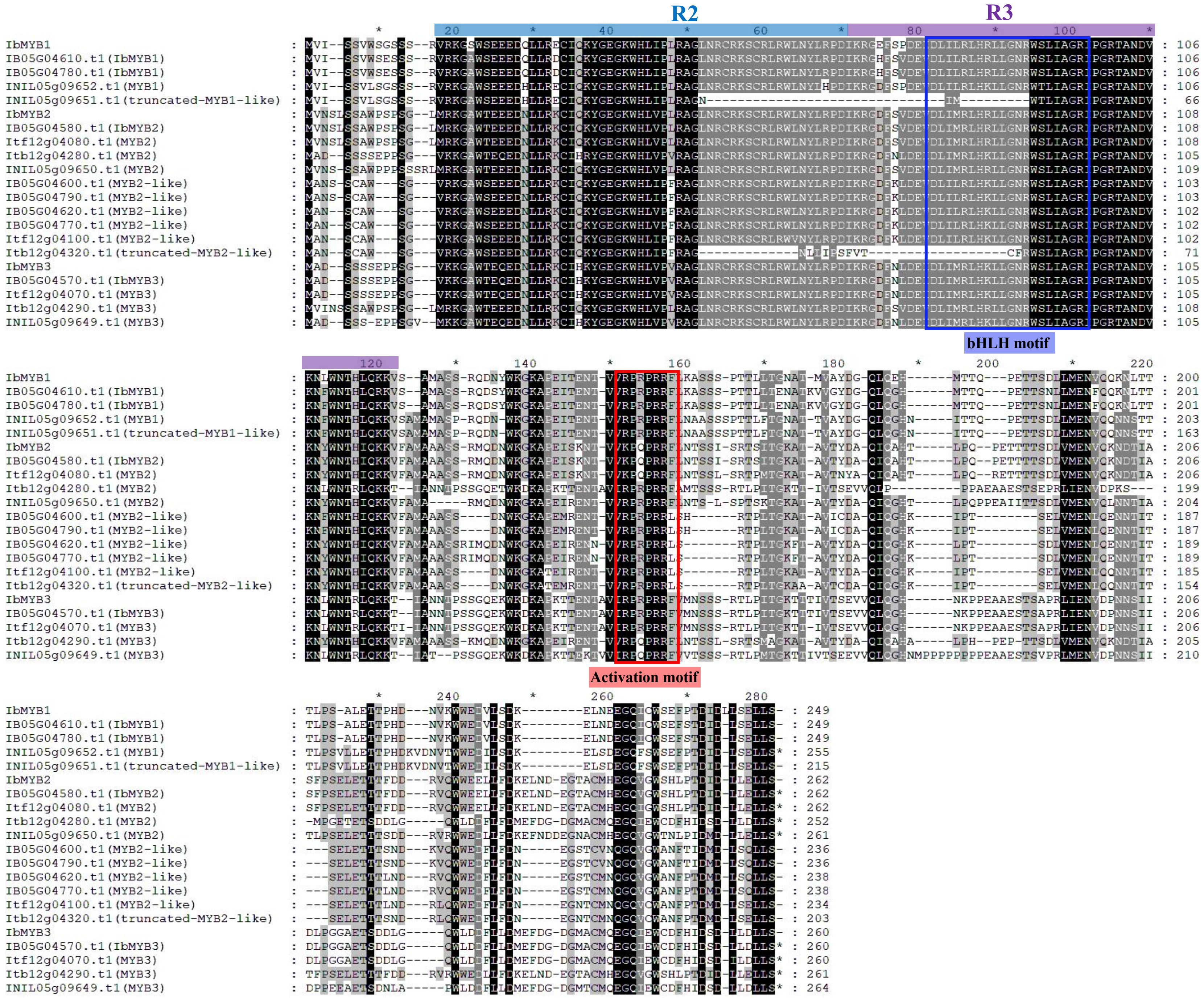

Figure S1. Multiple sequence alignment of IbMYB1, IbMYB2, IbMYB3, and their homologs in genus of *Ipomoea*.

Bars indicate R2 and R3 domains. The blue box highlights the motif interacting with bHLH, and the red box indicates the transcriptional activation motif.

Figure S2

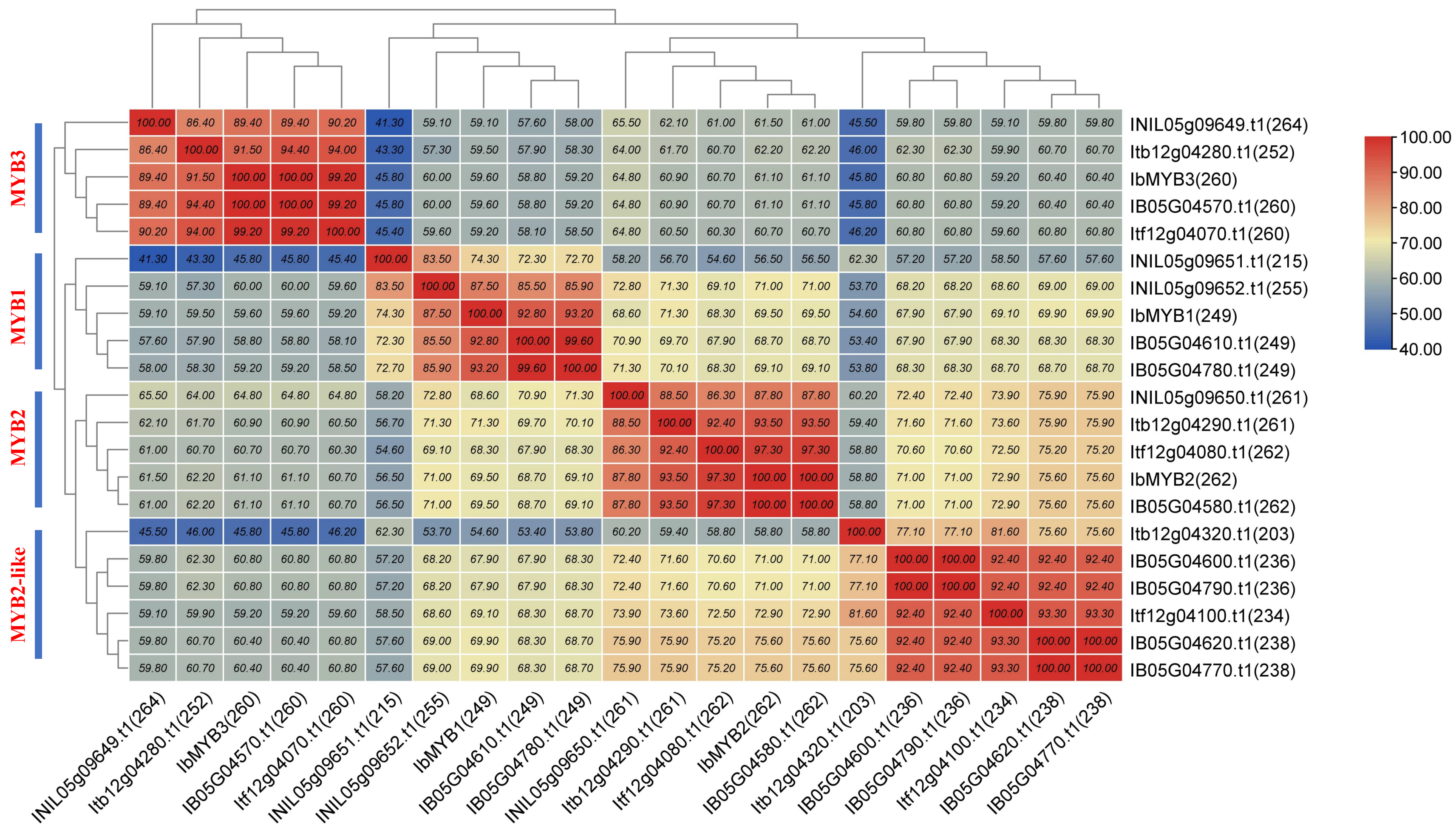

Figure S2 The identity between putative protein sequences of the *Ipomoea* MYBs..
The lengths of the proteins are indicated in brackets. Similarity categorized the *Ipomoea* MYBs into three branches: MYB1, MYB2, and MYB3. And the MYB2 branch splits into two groups: MYB2 and MYB2-like members

Figure S3

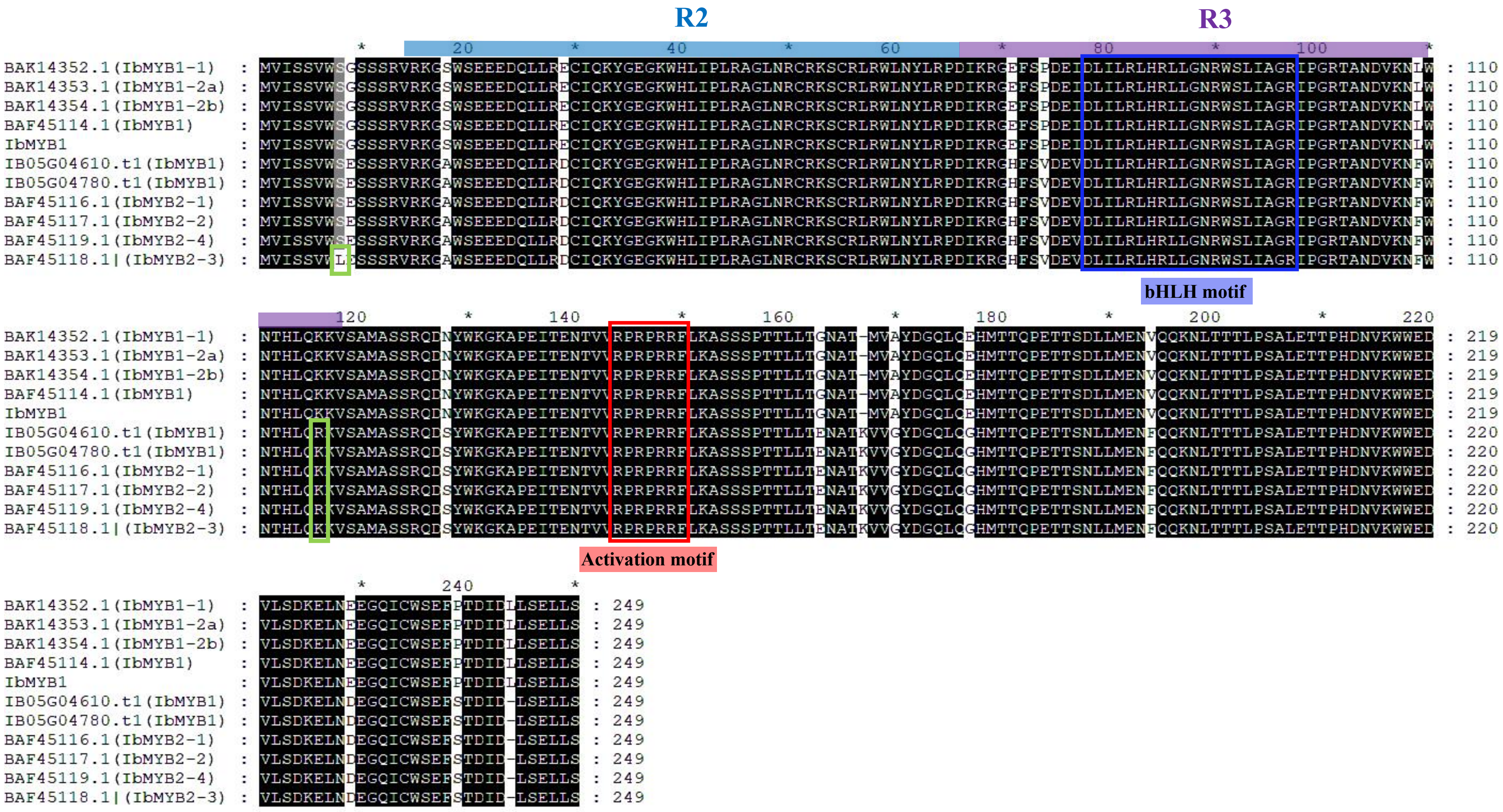

Figure S3. The multiple IbMYB1 and IbMYB2 isoforms from Mano et al. (2007) share high sequence identity.

The cloned IbMYB1 shows 100% protein sequence identity with IbMYB1 isoforms (BAK14352.1, BAK14353.1, BAK14354.1, and BAF45114.1). Identified IbMYB1 homologs in the "Taizhong 6" genome exhibit 99% protein sequence identity with IbMYB2 isoforms. The subtle differences are highlighted in green boxes.

Figure S4

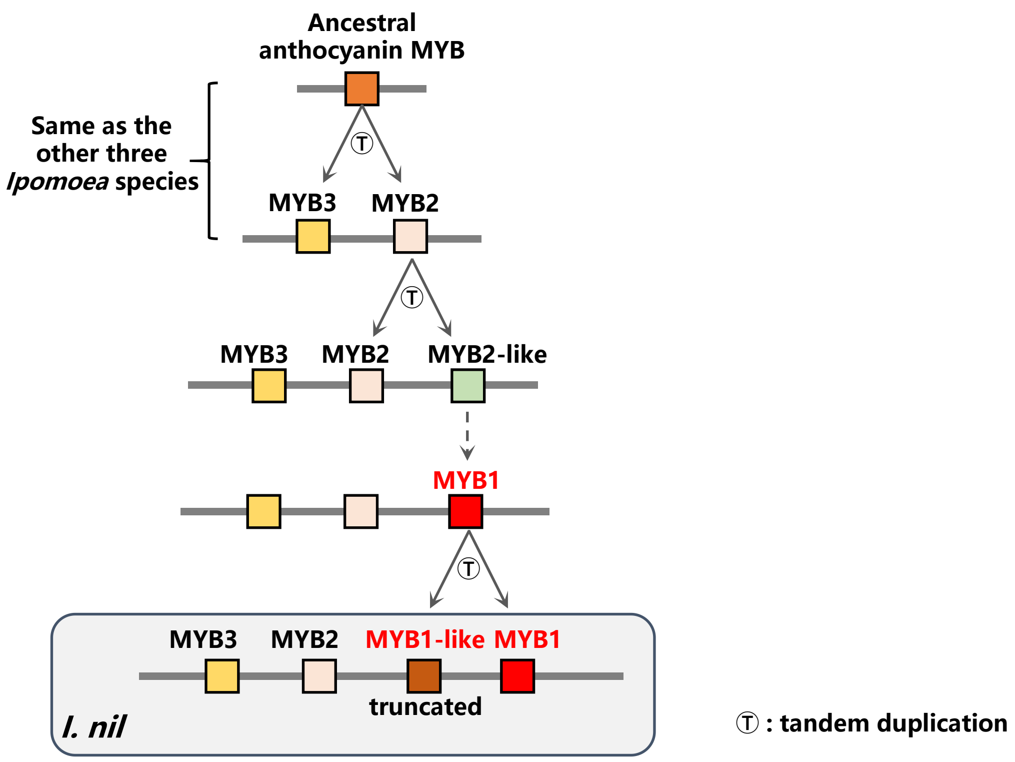

Figure S4. Schematic diagram of the hypothetical evolutionary scenario of MYB clusters in *I. nil*.

Figure S5

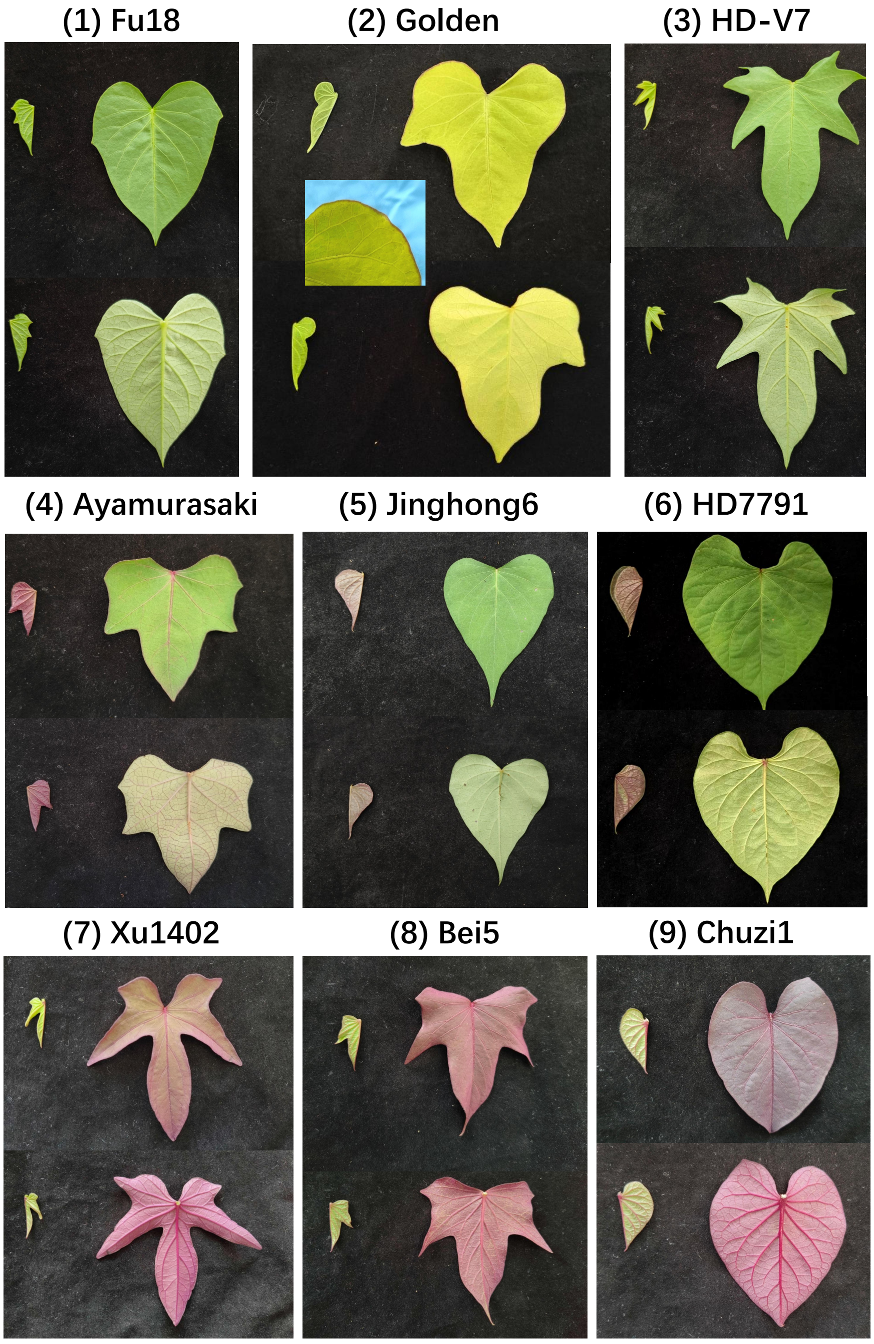

Figure S5. Enlarged presentation of leaves from nine representative varieties.
Top young leaves are shown on the left, and mature leaves on the right. Upper panels display the adaxial side, and lower panels show the abaxial side. The red leaf edge of "Golden" is highlighted in an inset image against a blue background.

Figure S6

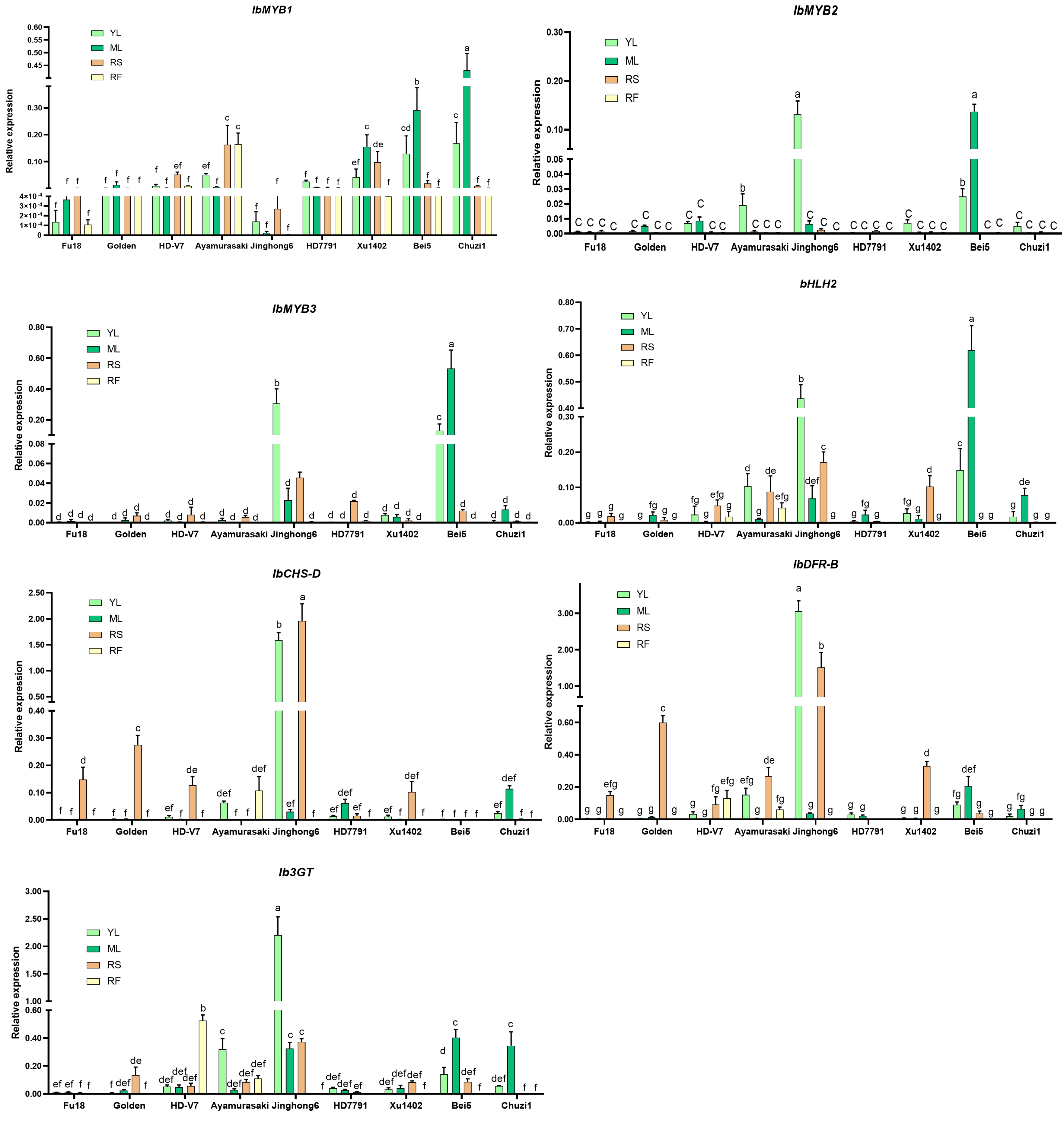

Figure S6. Expression analysis of IbMYB1/2/3 and key anthocyanin genes across sweetpotato varieties.

Relative expression was calculated using the ΔCt method relative to the reference genes. Differences among tissues and varieties for each gene were evaluated by one-way ANOVA ($p < 0.05$)

Figure S7

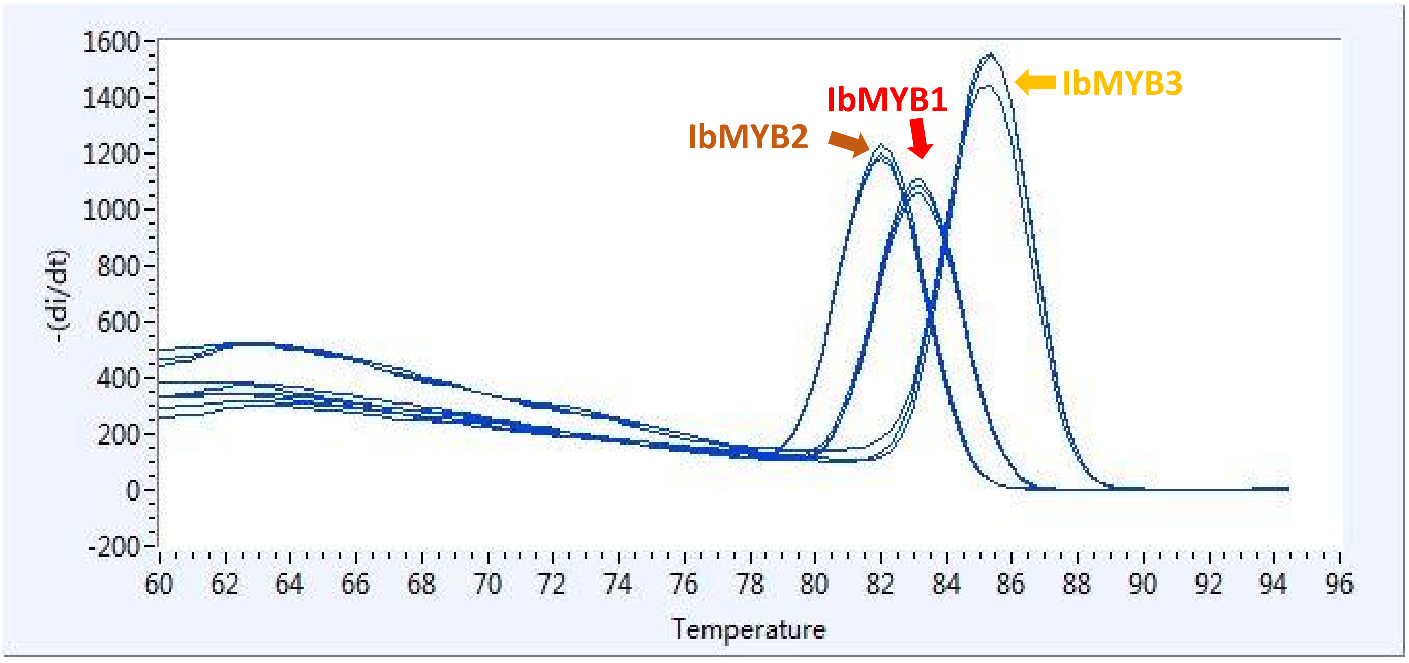

Figure S7. Dissolution curves of IbMYB1/2/3 amplified products using cDNA as templates in qRT-PCR.
Melting curves display single peaks for IbMYB1/2/3, indicating high amplification specificity.

Figure S8

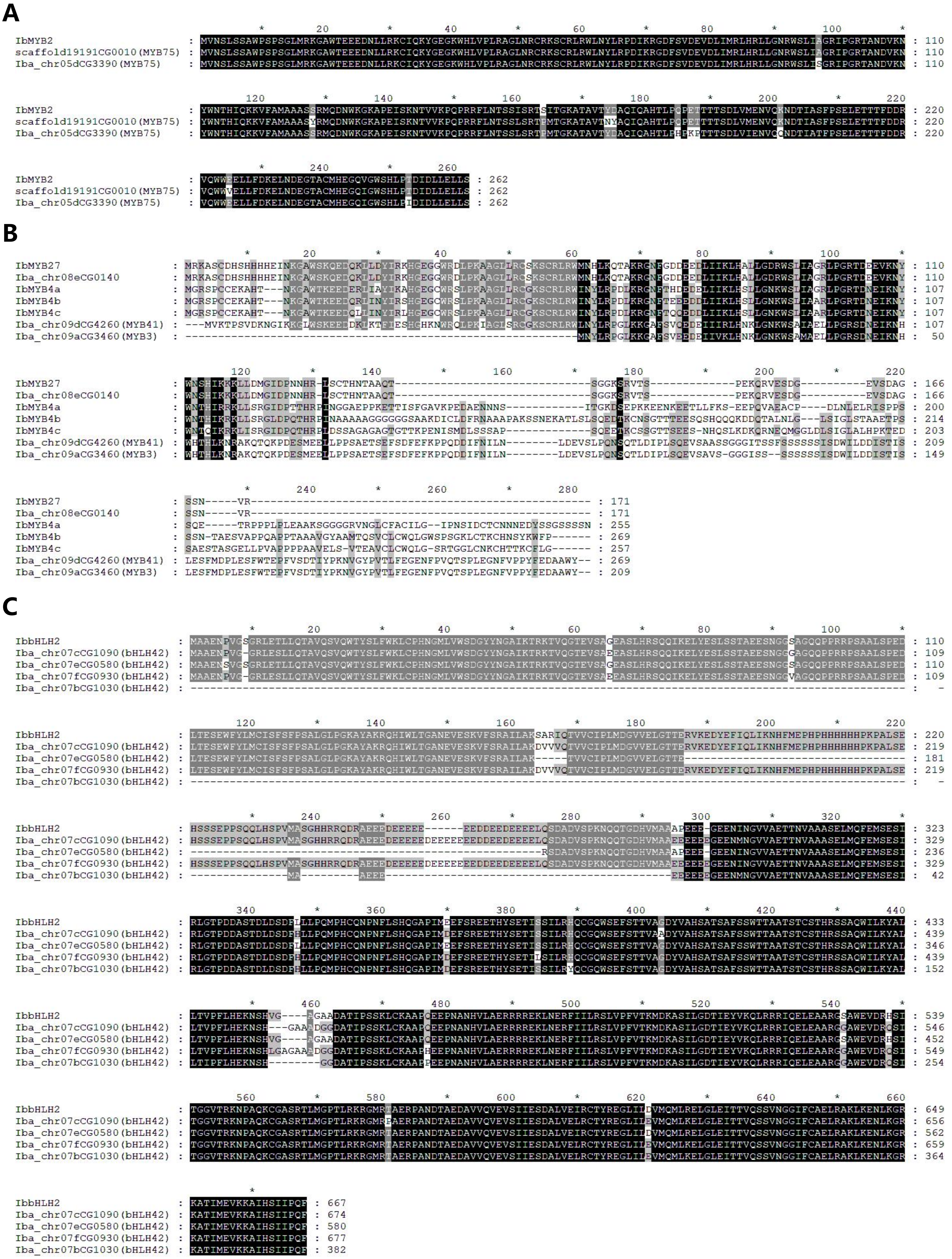

Figure S8. Protein sequence alignment of the top differentially expressed TFs with symbols or descriptions relevant to MBW members.
(A) Genes related to IbMYB2. **(B)** Genes related to IbMYB27, including two SG16 MYBs. **(C)** Genes related to IbbHLH2, with one showing a truncated N-terminal.
